# Shaping the Gut Microbiota by Breastfeeding: The Gateway to Allergy Prevention?

**DOI:** 10.3389/fped.2019.00047

**Published:** 2019-02-27

**Authors:** Lieke W. J. van den Elsen, Johan Garssen, Remy Burcelin, Valerie Verhasselt

**Affiliations:** ^1^School of Molecular Sciences, University of Western Australia, Perth, WA, Australia; ^2^Division of Pharmacology, Faculty of Science, Utrecht Institute of Pharmaceutical Sciences, Utrecht University, Utrecht, Netherlands; ^3^Institut National de la Santé et de la Recherche Médicale (INSERM), Toulouse, France

**Keywords:** breastmilk, allergy, gut microbiota, neonate, prevention

## Abstract

Evidence is accumulating that demonstrates the importance of the gut microbiota in health and diseases such as allergy. Recent studies emphasize the importance of the “window of opportunity” in early life, during which interventions altering the gut microbiota induce long-term effects. The neonate's gut microbiota composition and metabolism could therefore play an essential role in allergic disease risk. Breastfeeding shapes the gut microbiota in early life, both directly by exposure of the neonate to the milk microbiota and indirectly, via maternal milk factors that affect bacterial growth and metabolism such as human milk oligosaccharides, secretory IgA, and anti-microbial factors. The potential of breastmilk to modulate the offspring's early gut microbiota is a promising tool for allergy prevention. Here, we will review the existing evidence demonstrating the impact of breastfeeding on shaping the neonate's gut microbiota and highlight the potential of this strategy for allergy prevention.

## Why Breastfeeding as a Potential Strategy for Allergy Prevention by Microbiota Shaping?

Most of the human body is heavily colonized by all kinds of microorganisms, including bacteria, viruses, fungi, protozoa, and parasites. The largest amount of microorganisms are found within the colon, although the presence in other parts of the gastrointestinal tract cannot be neglected (1, 2). The development of the human gut microbiome is a highly complex process (1). The order and timing by which the gut is colonized early in life has a lasting impact on the microbiome and contributes largely to the variation in microbiota observed between individuals (3). Over the first 3 years of life, the microbiota evolves from relatively simple but rapidly increasing in diversity to an adult state that is more complex and more stable (1). In addition to mode of delivery and antibiotic exposure, nutrition is a key factor in shaping the early microbiota composition and function [as reviewed in Tamburini et al. (4)]. Besides the important role of the gut microbiota in nutrient and bile acid metabolism and the production of vitamins (5), colonization and signaling by microbes plays a pivotal role in gut mucosal immunity as well as systemic immunity. The development of the microbiota ecology parallels that of the gut mucosal immune system. Accumulating evidence is showing that perturbations in the gut microbiota in early life, while the immune system is still developing, can have long-lasting effects on local and systemic immune health.

To date, a clear protective effect of breastmilk on allergy development has not been demonstrated (6–11). However, breastmilk contains factors that can affect key players in allergy development such as gut barrier function, the gut microbiota and oral tolerance induction (12). Therefore, we propose that the modulation of breastmilk composition could be a promising tool for allergy prevention. Here we will review the existing evidence demonstrating the impact of breastfeeding on the neonate's gut microbiota and highlight the potential of shaping the neonate's gut microbiome through breastmilk to decrease allergic disease risk. A literature search was performed in PubMed on original studies and review articles addressing (1) the association of the early life gut microbiota with allergic outcomes (2) immunological mechanisms for the early life gut microbiota to affect allergy development and (3) how breastfeeding shapes the gut microbiota. The primary focus of the review were recent studies addressing these topics in the early life window.

## Modulating Early Life Gut Microbiota May Reduce Long-Term Allergic Disease Risk

### A Window of Opportunity to Alter the Gut Microbiota for Allergy Prevention

Epidemiological and animal studies have linked perturbations in the infant gut microbiota, when the immune system matures and the gut is colonized with microbiota, with disease risk later in life (4, 13). This highlights the existence of a window of opportunity for disease prevention, including atopic disease, which matches the period in life of breastfeeding. Germ-free (GF) mice have elevated levels of serum immunoglobulin (Ig)E because B cells undergo more isotype class switching to IgE in Peyer's patches and mesenteric lymph nodes. Microbial colonization of GF pups starting between birth and 1 week after weaning, completely inhibits this induction of IgE if a diverse microbiota is used. This implies the need for a critical level of microbial diversity following birth to prevent IgE induction (14). Colonization of GF mice with gut microbiota in early life, but not in adults, is also sufficient to protect from mucosal invariant natural killer T (iNKT) cell accumulation in the lung and allergic airway inflammation (15). Antibiotic administration exacerbated allergic airway inflammation, reduced FoxP3+ regulatory T cells (Treg) in the colon and increased serum IgE only when treatment was initiated in early life (16). In humans, a link between gut microbial dysbiosis in the first 100 days of life and an increased risk of asthma was demonstrated (17). Also other studies have shown an association between the early intestinal microbiota and the risk of allergic sensitization (18–20). All provide evidence of a link between early gut microbiota dysbiosis and an increased risk to develop allergic disease.

### Microbiota Richness, Diversity, Composition, and Metabolism in Early Life as Key Elements for Later Allergy Risk

It is still very preliminary to define a “healthy” or “normal” human gut microbiota. The most recent studies define a healthy microbiota as highly diverse, i.e., above 600.000 bacterial genes (21). Nutrition is certainly one way to reach such diversity (2, 22). In addition to diversity there is a core microbiota which is common the most human guts and which has been proposed as a “must have“-set of bacteria (23, 24). Their precise role still needs to be clearly defined but they are mostly involved in the control of inflammation and innate immunity. Although much less details are known in early life, more and more studies try to determine associations between alterations in neonatal microbiota and disease outcome. Microbiota richness, diversity, and composition all may play a role in shaping of the immune response by the gut microbiota. Gut microbial alterations are not limited to shifts in the abundance of certain microbes, they also include alterations in microbiota metabolism and changes in the production of microbial-derived metabolites such as short-chain fatty acids (SCFA) (25, 26).

Bisgaard et al. (18) demonstrated the inverse association between the early gut bacterial diversity and the risk of allergic sensitization, but not asthma or atopic dermatitis (18). However, another study showed that infants with cow's milk allergy had an increased diversity of the gut microbiota and an altered microbial composition dominated by Lachnospiraceae as compared to non-allergic infants (27). In a Chinese cohort, 20 key food allergy-associated bacterial genera, but no difference in fecal microbiota diversity, were observed. The specific microbiota signature detected, could distinguish between IgE-mediated and non-IgE-mediated food allergic infants. The genus *Clostridium sensu stricto* significantly correlated with antigen-specific IgE in infants with food allergy (28). A low gut microbiota richness, overrepresentation of Enterobacteriaceae and underrepresentation of Bacteroidaceae in early infancy were associated with food sensitization in a subset of the Canadian Healthy Infant Longitudinal Development (CHILD) study (20). In the same cohort, Canadian infants at risk of asthma showed a reduction in the relative abundance of the bacterial genera *Lachnospira, Veillonella, Faecalibacterium*, and *Rothia* in early life and had lower fecal concentrations of the SCFA acetate (17, 29). A causal role of these bacterial taxa was demonstrated in mouse experiments (17). The impact of microbial dysbiosis at 3 months of age was further confirmed in a non-industrialized population in rural Ecuador (30). Interestingly, different bacterial taxa were involved compared to Canadian infants. Some fecal fungal taxa were altered too and genes involved in carbohydrate and taurine metabolism were highly altered (30). Another birth cohort showed that neonates with a relatively lower abundance of bacteria such as *Bifidobacterium, Akkermansia*, and *Faecalibacterium*, along with higher abundance of the fungi *Candida* and *Rhodotorula* and pro-inflammatory fecal metabolites, had the highest risk of childhood atopy and asthma (31). Russian children at low risk for the development of allergic disease had higher proportions of *Bifidobacterium*, whereas Finnish and Estonian children with a higher risk of allergies had increased abundance of *Bacteroides* (32). Furthermore, early colonization with Lactobacilli was shown to decrease the risk of allergy (19) while early colonization with *Staphyloccocus aureus* and *Clostridium difficile* characterizes infants developing allergy later in life (33–35). Colonization with *Escherichia coli* was associated with IgE-mediated eczema (36, 37). However, a study using early administration of *E. coli* as probiotic strategy found a reduction in allergy development, pointing toward strain-specific effects of *E. coli* (38). Recently it was also reported that the kinetic of development of the gut microbiome during the first year of life affects the risk of childhood asthma in children from asthmatic mothers. One-year-old children with an immature microbial composition had an increased risk of asthma at age 5 years compared to children with mature microbiota (39).

## Potential Mechanisms of Allergy Prevention in Early Life by the Microbiota

**Figure 1:**
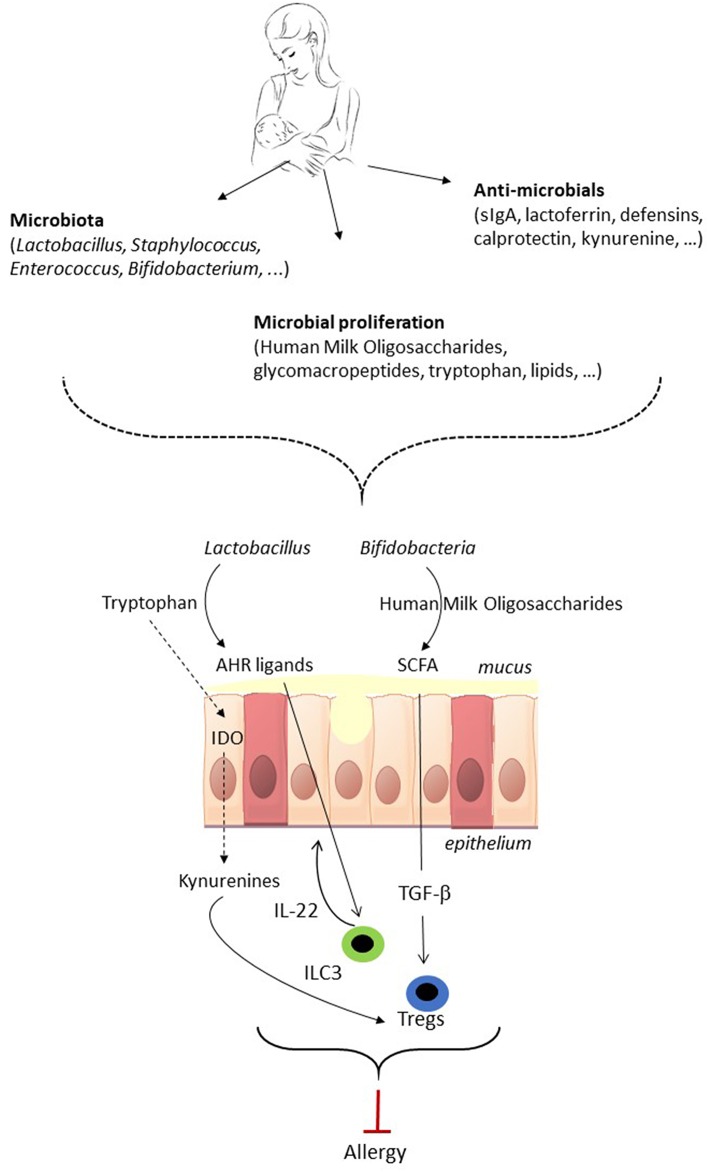
The potential of breastmilk to prevent allergic disease by shaping of the neonatal gut microbiota. Breastmilk contains microbes as well as factors that indirectly shape the gut microbiota of the neonate. Breastmilk can direct the early microbiota composition, i.e., favor the growth of *Bifidobacteria* and *Lactobacillus*, and affect microbiota metabolic function, which subsequently can impact on immune development and maturation. The gut microbiota in early life impacts on immune maturation via microorganism-associated molecular patterns (MAMPs) signaling (not shown in the figure) and via microbiota metabolites such as short-chain fatty acids (SCFA) and aryl hydrocarbon receptor (AHR) ligands. The gut microbiota of the neonate can direct the immune system toward allergy prevention via the induction of FoxP3+ regulatory T cells (Treg), which contribute to oral tolerance induction, and IL-22 production by group 3 innate lymphoid cell (ILC3) which strengthens the gut barrier. As a result, shaping of the infant's gut microbiota by breastmilk has the potential to direct the immune system toward allergy prevention. TGF-β, Transforming Growth Factor beta; IDO, indoleamine 2,3-dioxygenase.

To induce tolerance at mucosal surfaces, antigens are taken up by dendritic cells (DC) which migrate to the lymph nodes where the local production of factors like transforming growth factor beta (TGF-β) induces the differentiation of naïve T cells to antigen-specific Treg (40). Here, we will summarize the current observations in early life specifically, as this coincides with the period of breastfeeding, which demonstrate an effect of the microbiota on the maturation of the immune system (Figure 1). Various studies have demonstrated a role of the microbiota in early life on the development of FoxP3+ Treg. *Ex vivo* culturing of human adult peripheral T cells with sterile fecal water from children at high risk of developing atopic disease, reduced the percentage of FoxP3+ Treg cells (31). Neonatal colonization with a specific strain of the commensal *E. coli* lead to oral tolerance failure. It reduced tolerogenic DC and subsequently Treg populations (41). On the other hand, neonatal enrichment of mice with *Clostridium* species from human indigenous microbiota resulted in higher numbers of colonic FoxP3+ Treg in adulthood, likely induced by intestinal epithelial cell-secreted TGF-β, and lower allergy risk (42). Another study demonstrated the pivotal role of early life colonization with *Bacteroides fragilis* expressing polysaccharide A (PSA) for iNKT cell inhibition and Treg development in the intestine (43). Colonizing adult mice did not have this effect (43). Another study has emphasized a role for the gut microbiota in the modulation of IL-22 secretion and gut barrier function. Colonization of young mice with Clostridia induced IL-22 production by group 3 innate lymphoid cells (ILC3) and T helper 17 cells in the intestinal lamina propria. IL-22 was critical for sensitization to food allergen as it induces antimicrobial peptide production by Paneth cells and mucus production by goblet cells to strengthen the gut barrier. This prevents the transfer of dietary antigen across the barrier and therefore allergic sensitization (44). Recent studies have also linked the kynurenine pathway, involved in the breakdown of tryptophan by host cells, with the gut microbiota and allergy (45). In host immune and epithelial cells the enzyme indoleamine 2,3-dioxygenase (IDO) metabolizes tryptophan to kynurenine and downstream products (46). IDO can become activated in response to allergen-induced immune activation (45). Kynurenines regulate immune homeostasis and exhibit tolerogenic effects by causing T cell anergy and apoptosis and induce the generation of Treg, leading to attenuation of allergic responses (45). The gut microbiota plays an important role in stimulating IDO activity, as demonstrated in GF mice (46, 47), making this pathway dependent on early life gut microbiota development (45). Targeting the gut microbiota to modulate tryptophan metabolism could therefore have the potential to prevent allergic disease (45).

### Shaping of the Immune System by the Microbiota

#### MAMP Signaling

The gut microbiota exerts direct effects on the immune system through microorganism-associated molecular pattern (MAMP) signaling. Bacterial as well as fungal and viral molecular patterns such as lipopolysaccharides (LPS), flagellin, peptidoglycans (PG), formyl peptides, and unique nucleic acid structures are sensed by pattern recognition receptors (PRR) including membrane bound Toll-like receptors (TLR) and cytoplasmic NOD-like receptors (NLR) (25).

LPS binds to a complex of membranous TLR4, CD14 and myeloid differentiation protein 2 (MD-2) or circulating sCD14 receptors. TLR4 is the receptor transmitting the MAMP's signal inside the cell to trigger genes of inflammation. LPS are complex molecules with a lipid A part containing different fatty acids esterified to a glucosamine structure. It can possess 4, 5, or 6 aliphatic chains of different length including cyclic fatty acids specific from bacteria. Hexa-acylated LPS, such as seen in *E. coli*, is considered highly inflammatory whereas LPS with a lower number of acylated fatty acids, such as those from the Porphyromonadaceae family, are less inflammatory or even anti-inflammatory (48). Variation in LPS immunogenicity can play a role in immune education and driving allergic disease. High exposure to *Bacteroides*-derived penta- or tetra-acylated LPS, which has immune inhibitory properties, was demonstrated in infants with high risk of allergic disease. This form of LPS does not induce endotoxin tolerance, leading to inflammatory responses later in life (32, 49). A similar observation was reported for PGs which can be pro- or anti-inflammatory according to the structure and molecular weight of the molecule. In addition, PG can bind to different TLRs, particularly TLR2, as well as the intracellular NLR NOD1 and 2, with different outcome (50, 51). Furthermore, TLR signaling by bacterial products induces a tolerogenic environment and Treg expansion in the intestine (52). These studies demonstrate that MAMPs can engage innate and adaptive immunity through the stimulation of different TLRs and NLRs. This suggests that the gut microbiota ecology at birth directs the specific crosstalk between MAMPs and the immune system and educates both innate and adaptive immunity. This could result in protection against the development of allergic diseases.

#### Metabolite Signaling

Besides the direct effects of gut microbes on the immune system, the production of metabolites by the fermentation of dietary fiber and other complex macronutrients that escape digestion in the small intestine, plays an important role. Molecules produced or derived from bacterial metabolism are drivers of cellular host functions and notably the intestinal immune, epithelial, vascular, and neural systems (53). Bacterial metabolites can induce epigenetic changes such as chromatin modifications that allow the microbiota to exert long-lasting effects on immunity (54). Metabolites of microbial origin include choline metabolites, vitamins, and phenolic derivatives (53). Primary bile acids can be transformed into secondary bile acids by the metabolism of bacteria, which can differently activate the bile acid receptors farnesoid X receptor (FXR) and G protein-coupled bile acid receptor (TGR5) (55). Specific commensal bacteria, especially *Lactobacilli*, metabolize the essential amino acid tryptophan into indole derivatives that can bind to aryl hydrocarbon receptors (AHR) expressed by immune and epithelial cells. AHR signaling is important for ILC3 activation and intestinal barrier function (25). The role of AHR ligands on immune development during the neonatal phase specifically is largely unclear to date. Branched-chain fatty acids such as valerate, isobutyrate, and isovalerate are derived from bacterial amino acid metabolism. SCFA, predominantly butyrate, acetate and propionate, are produced upon breakdown of dietary fiber. Lactate and succinate are intermediate metabolites in the production of SCFA, but can exert immune modulating effects themselves too (56, 57). SCFA are an important energy source for intestinal epithelial cells. Furthermore, SCFA signal through G protein couples receptors such as GPR43, GPR41, and GPR109A present on epithelial and immune cells, and inhibition of histone deacetylases (25, 58). In adult mice it has been demonstrated that SCFA can enhance the intestinal barrier function, induce tolerogenic DC and promote anti-inflammatory Treg in the colon, all contributing to immune tolerance (25). In the lung SCFA impair the ability of DC to promote a T helper 2 response and reduce ILC2 proliferation and function (59, 60). Data on the effects of SCFA on the maturation of the gut and immune system in the neonate is currently largely lacking. However, there is limited evidence that higher acetate levels in infants might assist in the protection against allergic disease (17).

## Breastfeeding Shapes Gut Microbiota Composition and Metabolism

### A Role of Breastmilk in the Establishment of the Gut Microbiota in the Neonate

Recent studies suggest that microbial transfer from the mother to the fetus already occurs *in utero*. Microbes have been detected in the placenta, amniotic fluid, fetal membrane, umbilical cord blood, and meconium (4, 61). However, the first major exposure of the neonate to microbes happens during birth and is highly dependent on the mode of delivery (1, 4). Besides birth mode and/or antibiotics use just before or after delivery, early nutrition is a key factor directing the early microbiota composition and function as it provides nutrients for bacterial growth and dictates their production of metabolites (1, 2, 22). Recently, a large, multi-center study confirmed that breastfeeding status was the most significant factor associated with microbiome structure in early life (62). The first bacteria to establish in the neonatal gut are mostly aerobic or facultative anaerobic bacteria such as enterobacteria, enterococci, and staphylococci. During their growth they consume oxygen allowing the rise of anaerobic bacteria including bifidobacteria (63). The gut microbiome in breastfed infants is usually dominated by bifidobacteria and *Lactobacillus* species, while formula-fed infants harbor a more diverse gut microbiota that resembles that of older children (22, 64). Relatively small amounts of formula supplementation of breastfed infants, only during the first days of life, already resulted in shifts in microbiota composition (65). In addition to shaping microbiota composition, early feeding practice affects microbiota metabolism. The microbiomes of newborns and young infants are enriched in genes required for the degradation of sugars from breastmilk (human milk oligosaccharides, HMOs) (22). Compared with formula-fed children, breastfed infants have lower absolute concentrations of fecal SCFA, potentially due to the less diverse microbiota, and higher concentrations of lactate. However, the relative proportion of acetate was higher in exclusively breastfed children (56). The introduction of solid foods changes the metabolic function of the gut bacteria as genes involved in the degradation of sugars from breastmilk are less needed and utilized. Instead, the microbiota adapts to the available energy sources and functionally matures to be able to degrade complex sugars and starch found in solid food (22). Interestingly, the microbiota composition in African and European infants is very similar until the introduction of solid foods, indicating the dominant role of diet over other variables in shaping the microbial composition of the gut in early life (66).

### Mechanisms of Breastmilk-Induced Neonatal Gut Microbiota Shaping

Breastmilk provides the neonate with its own microbiota as well as prebiotic, immunological and other microbiota-shaping compounds that indirectly can alter colonization patterns in the neonate (Figure 1). Therefore, a varied composition of breastmilk could be considered as a selective bioactor to reach gut microbiota diversity and hence good health.

#### Human Breastmilk Microbiota

Breastmilk contains 10^2^-10^4^ viable bacteria per mL (67), and thereby can directly affect the establishment of the neonatal microbiota (68). *Lactobacillus, Staphylococcus, Enterococcus*, and *Bifidobacterium* are transferred through breastfeeding (67). Milk bacterial communities are complex and vary between individuals. The breastmilk microbiota also evolves over the period of breastfeeding. Colostrum microbiota has a higher diversity than mature milk (69). In colostrum, *Staphylococcus*, lactic acid bacteria and *Streptococcus* are the most abundant (69). After 1 month, the *Staphylococcus* abundance is dramatically reduced, while the lactic acid bacteria are still highly abundant (68, 69). The maturation of the breastmilk microbiota happens in parallel with the evolution of the neonate's microbiota. As soon as 3–4 days after birth, the gut microbiota of infants begins to resemble the colostrum microbiota (61), followed by a gut microbiota rich in bifidobacteria and lactobacilli (22).

The origin of bacteria in breastmilk is under debate. Some suggest that human milk bacteria are derived from the maternal skin as some bacterial phyla that are common in human milk, such as *Staphylococcus*, are usually present on adult skin (67). It has also been demonstrated that during suckling breastmilk flows back into the mammary ducts (67, 70), which provides a route for bacteria found in the infants oral cavity to enter the mammary gland (67, 69). However, most studies propose that the translocation of maternal gut bacteria to the mammary gland is the major pathway (2, 64). DC and macrophages can sample live commensal bacteria from the gut lumen and keep them in the mesenteric lymph nodes. From there, the bacteria can circulate to other locations in the body, including the mammary glands (67). Mothers having a cesarean section, show a more diverse milk microbiota with reduced frequency of bifidobacteria as compared to mothers after a vaginal delivery. This effect is most pronounced for infants from women undergoing an elective cesarean-section, suggesting that signals related to labor affect bacterial transfer to the mammary glands (69).

#### Human Milk Oligosaccharides

HMOs are structurally complex sugars unique to human breastmilk. They are indigestible and do not provide energy for the infant but serve as prebiotics, which are substrates for fermentation processes by intestinal microbes, inducing the growth or activity of beneficial bacteria (71). HMOs are highly abundant in human milk but absent in most formula nutrition and are believed to play a major role in the differences between the gut microbiota in breast- vs. formula-fed infants. HMO composition in maternal milk is regulated by genetic fucosyltransferase-2 (FUT2) secretor status and other factors including lactation stage, maternal health and ethnicity (72). HMOs act as antiadhesive agents that inhibit pathogen adhesion to mucosal surfaces, preventing colonization and as antimicrobials by preventing proliferation of certain bacteria (72, 73). Furthermore, HMO favor *Bifidobacterium* growth and are their preferred substrates for the production of SCFA and lactate in infancy (56, 74, 75). *Bifidobacterium* predominance in the stool is the main characteristic of breastfed infants (68) and the higher relative proportion of acetate in breastfed compared to formula-fed infants may be due to the absence of HMO in formula (56). Infants receiving breastmilk from non-secretor mothers, who lack the functional FUT2 enzyme, show a delay in the establishment of bifidobacteria highlighting the need for maternal milk HMOs in bifidobacteria growth (76). A recent study further demonstrates that HMOs in breastmilk can also modulate the transcriptional activity of certain bacteria such as *B. fragilis*, rather than modifying their relative abundance (26). Furthermore, another commensal, *E. coli*, that cannot directly degrade HMOs, benefits indirectly by consuming metabolites produced by *B. fragilis* upon HMOs degradation (26). An association between HMO profiles, but not individual HMOs, and food sensitization has been demonstrated in 1-year-old infants (77). A small clinical study further found that infants receiving maternal milk with low concentrations of the HMO lacto-N-fucopentaose III were more likely to develop cow's milk allergy (76). A likely explanation, besides the possibility of direct effects of HMO on immune cells, is the prebiotic modulation of the gut microbiota by HMOs influencing immune development and food sensitization (77).

#### Glycomacropeptide

Casein glycomacropeptide (GMP) is a small glycoconjugated peptide present in human milk. GMP has a prebiotic effect on bifidobacteria and lactic acid bacteria (78).

#### Secretory IgA

Secretory IgA (sIgA) are also important factors in shaping the gut microbiota in the neonate. IgA-producing plasma cells in the mammary gland originate from the maternal gut. The specificity of sIgA in breastmilk is therefore dictated by maternal exposure to pathogenic enteric bacteria and by commensal bacteria in the maternal gut. Maternal IgA produced in the mammary gland, are transported across the epithelial cells into the milk and acquire the secretory part from epithelial cells (79). As newborns produce only low levels of sIgA, breastmilk-derived sIgA prevent expansion and penetration of pathogenic bacteria while their intestinal immune system is developing (80, 81). IgA also has functions beyond pathogen exclusion. Experiments in mice using IgA-deficient dams demonstrated a long-lasting role of breastmilk-derived sIgA in shaping of the gut microbiota. Taxa from the family Lachnospiraceae were upregulated in the absence of sIgA from breastmilk (82). Milk sIgA are also necessary for the prevention of excessive expansion of pro-inflammatory segmented filamentous bacteria (SFB) by coating these bacteria (83). A recent birth cohort showed that IgA recognition patterns differed between healthy and allergic children. This was already visible at 1 month of age, when IgA is predominantly maternally derived in breastfed children. Interestingly, mainly butyrate-producing gut commensals such as *Faecalibacterium* were IgA free in children with allergic symptoms (84). IgA can also modulate the production of metabolites by the microbiota, as recently shown in an adult mouse model (85). In this study it was demonstrated that IgA has the capacity to modulate bacterial composition, gene expression, and metabolic function of the gut microbiota by antigen-independent binding to intestinal bacteria (85).

#### Antimicrobial Proteins and Peptides

Antimicrobial factors in breastmilk have the potential to shape the microbiota. Breastmilk is the main source of lactoferrin for the infant and protects them from bacterial invasion by sequestering iron from bacterial pathogens and direct interaction with bacteria (79). The ability to protect the infant against pathogenic microorganisms helps the development of a beneficial microbiota (79). The amount of fecal bifidobacteria and lactobacilli in newborns positively correlated with fecal lactoferrin (86). This suggests that lactoferrin promotes specific microbial composition and might be critical for the microbiota to develop in early life. High levels of calprotectin (heterodimer of calcium binding proteins S100A8 and S100A9) also demonstrate antimicrobial properties that can be attributed to its metal ion chelation capacity. This results in growth inhibition of especially manganese sensitive bacteria such as *S. aureus* and group B streptococci. After birth, calprotectin concentrations are high in human milk (87). Defensins in breastmilk also have antimicrobial activity against common neonatal pathogens (88) and have been shown to affect the intestinal microbiota (89).

#### Tryptophan Metabolites

Tryptophan is a precursor of a large number of metabolites, including endogenous metabolites such as kynurenine and bacterial metabolites such as indole derivatives (46, 47). Tryptophan and its metabolites are present in breastmilk (90, 91) and can have profound effects on the gut microbial composition, metabolism and function in the infant (47). Kynurenines have antimicrobial properties, which can directly impact on the gut microbiota (47). AHR ligands in breastmilk originate from the maternal diet as well as from the maternal microbiota. Maternal milk immunoglobulins help in the transfer of these metabolites to the neonate (91). The AHR signaling pathway is also able to influence microbiota composition (92).

#### Components of the Innate Immune Response

Factors of the innate immune response that are present in breastmilk, but not infant formula, include soluble TLR2, TLR4 and their co-receptors CD14 and MD2, which are involved in binding LPS (89, 93, 94). These factors probably contribute to the composition of luminal and enterocyte surface bacteria (89, 95).

#### Lipids

Lipids impact on the gut microbiota ecology either directly by feeding some bacteria or indirectly by triggering the host to secrete hormones and bile acids (96). Bile acids are major regulators of the gut microbiota (55). They are detergents for bacteria explaining, at least in part, the low bacterial count in the duodenum where bile acids are mostly released. Human milk fatty acid profiles were associated with the relative abundance of five taxa (*Bacteroides*, Enterobacteriaceae, *Veillonella, Streptococcus*, and *Clostridium*) in the gut microbiota of breastfed neonates (97).

## Conclusions and Perspectives

The potential of breastmilk to alter the offspring's early gut microbiota is a promising tool for immune education and allergy prevention (Figure 1). This requires identifying (1) what a beneficial microbiota for allergy prevention in our modern environment is (2) which factors in breastmilk are necessary to guide the establishment of such a beneficial microbiota and (3) how to enrich breastmilk with the required factors. There have been major advances in the recent years in the identification of a beneficial microbiota for allergic disease prevention. In particular, the necessity of a diverse gut microbiota and the importance of the metabolism of the microbiota have been highlighted. The role of the breastmilk microbiota and HMOs in shaping of the neonatal gut microbiota has become more and more evident recently. There is however still poor knowledge on the possibilities to modulate breastmilk factors involved in the microbiota shaping. The administration of probiotics to lactating mothers has led to inconsistent results regarding the possibility to change the milk microbiota (98, 99) and there is currently no clue on the possibility to modify HMOs content in breastmilk. There is a need for randomized intervention trials that study the allergy preventive effects of supplementation of lactating mothers with milk-modulating factors such as pre- and probiotics, to shape the infants microbiota and subsequently program the immune response.

## Author Contributions

LvdE and VV proposed the topic to be covered in the manuscript and wrote the main content with the contribution of JG and RB.

### Conflict of Interest Statement

JG is employed by Danone, a company producing products for infant nutrition. The remaining authors declare that the research was conducted in the absence of any commercial or financial relationships that could be construed as a potential conflict of interest.
